# Serum Inter-Alpha-Trypsin Inhibitor Heavy Chain 4 (ITIH4) in Children with Chronic Hepatitis C: Relation to Liver Fibrosis and Viremia

**DOI:** 10.1155/2014/307942

**Published:** 2014-09-14

**Authors:** Mostafa M. Sira, Behairy E. Behairy, Azza M. Abd-Elaziz, Sameh A. Abd Elnaby, Ehab E. Eltahan

**Affiliations:** ^1^Department of Pediatric Hepatology, National Liver Institute, Menofiya University, Shebin El-koom, Menofiya 32511, Egypt; ^2^Department of Microbiology and Immunology, National Liver Institute, Menofiya University, Shebin El-koom, Menofiya 32511, Egypt; ^3^Department of Pediatrics, Faculty of Medicine, Menofiya University, Shebin El-koom, Menofiya 32511, Egypt

## Abstract

Liver fibrosis and viremia are determinant factors for the treatment policy and its outcome in chronic hepatitis C virus (HCV) infection. We aimed to investigate serum level of inter-alpha-trypsin inhibitor heavy chain 4 (ITIH4) and its relation to liver fibrosis and viremia in children with chronic HCV. ITIH4 was measured by ELISA in 33 treatment-naive children with proved chronic HCV and compared according to different clinical, laboratory and histopathological parameters. Liver histopathological changes were assessed using Ishak score and compared with aspartate transaminase-to-platelet ratio (APRI) and FIB-4 indices as simple noninvasive markers of fibrosis. ITIH4 was measured in a group of 30 age- and sex-matched healthy controls. ITIH4 was significantly higher in patients than in controls (54.2 ± 30.78 pg/mL versus 37.21 ± 5.39 pg/mL; *P* = 0.021). ITIH4, but not APRI or FIB-4, had a significant direct correlation with fibrosis stage (*P* = 0.015, 0.961, and 0.389, resp.), whereas, the negative correlation of ITIH4 with HCV viremia was of marginal significance (*P* = 0.071). In conclusion, ITIH4 significantly correlated with higher stages of fibrosis indicating a possible relation to liver fibrogenesis. The trend of higher ITIH4 with lower viremia points out a potential antiviral properties and further studies in this regard are worthwhile.

## 1. Introduction

Hepatitis C virus (HCV) infection is a serious health problem that may result in chronic hepatitis, cirrhosis, and hepatocellular carcinoma. It is estimated that over 200 million people are infected worldwide, while 80% develop a chronic form [1]. HCV prevalence varies geographically, with rates of 1.7% in the United States, 2.1% in Southeast Asia, and 5.3% in Africa [2]. In children younger than 11 years, worldwide seroprevalence of HCV is 0.2% and in those older than 11 years it is 0.4% [3].

Egypt reports the highest prevalence worldwide ranging from 8.7% in upper Egypt to 24.3% in lower Egypt with genotype 4 in more than 90% of those infected [4]. Studies of the magnitude of HCV infection in Egyptian children revealed a prevalence of 3% in upper Egypt and 9% in lower Egypt [5]. Liver disease seems to be milder in children than in adults; however, the natural history of HCV infection acquired in infancy and childhood remains poorly characterized and the long-term outcome of the disease is still a matter of debate [2].

Although liver biopsy represents the gold standard for evaluating presence, type, and stage of liver fibrosis and for characterizing necroinflammation, it remains an invasive procedure with inherent risks. Thus, it cannot be performed frequently to monitor therapeutic outcomes [6, 7]. Moreover, in children, biopsy is still perceived to carry a higher risk of complications, so it is less accepted than in adults. Therefore, developing noninvasive tests that can predict initial disease stage and progression over time represents a high priority and a growing medical need [8, 9].

A recent study, using proteomic analysis of serum from adult patients with chronic HCV infection, revealed that inter-alpha-trypsin heavy chain 4 (ITIH4) was a candidate to predict liver fibrosis [10]. ITIH4 was found to be higher in HCV patients with mild to moderate fibrosis compared to healthy controls, while in those with cirrhosis, the net production of ITIH4 was found to be downregulated [11].

ITIH4 is a plasma glycoprotein that is expressed mainly in the liver [12]. It is one of the inter-alpha-trypsin inhibitors (ITI) family which are found in human plasma in relatively high concentrations. As their original names suggest, the family molecules were studied extensively as protease inhibitors [13]. It was reported that influenza viral replication was inhibited by the protease inhibitor; trypsin inhibitor [14].

We aimed to investigate serum level of ITIH4 and its relation to liver fibrosis and viremia in children with chronic HCV infection.

## 2. Materials and Methods

### 2.1. Study Population

The study included 33 children with proved chronic HCV infection recruited from the outpatient and inpatient of Pediatric Hepatology department, National Liver Institute, Menofiya University. Diagnosis of chronic hepatitis C was based on the presence of serum anti-HCV antibody (Ab) and persistently positive HCV-RNA as detected by polymerase chain reaction (PCR) for more than 6 months [15, 16], negative hepatitis B viral markers, and absence of any associated liver disease, supported by the histopathological feature of HCV infection in liver biopsy. A second group of 30 healthy children with no signs or symptoms of liver disease or any other diseases, normal liver transaminases, and negative anti-HCV Ab served as controls. A signed informed consent was obtained from the legal guardians of all the patients and controls before enrollment in the study. The study was approved by the Research Ethics Committee of the National Liver Institute.

### 2.2. Laboratory Investigations

Laboratory investigations, including liver function tests, complete blood count (CBC), kidney function tests, serum autoantibodies (anti-nuclear antibodies, anti-smooth muscle antibodies, and liver-kidney microsomal antibodies) and prothrombin time were performed for all the patients. Serum viral markers were performed using enzyme-linked immunosorbent assay (ELISA) according to the manufacturer instructions, HCV Ab (Innogenetics, Ghent, Belgium), hepatitis B virus surface antigen, hepatitis B virus core immunoglobulin (Ig)M, and IgG Abs (all from Dia Sorin, Saluggia, Italy). Real-time PCR for HCV-RNA was performed using COBAS Ampliprep/COBAS TaqMan, Roche Molecular Systems, Inc., Branchburg, NJ, 08876 USA (detection limit was 15 IU/mL). According to the viral load, viremia was classified arbitrarily into low (<2 × 10^5^ IU/mL), moderate (≥2 × 10^5^ –2 × 10^6^ IU/mL), and high viremia (≥2 × 10^6^ IU/mL) [17]. Serum ITIH4 levels were assayed using ELISA kit (WKEA Med Supplies Corp, NY 10123, United States) according to the manufacturer instructions. Serum samples of the patients were collected, maximally, within 6 months of liver biopsy [18]. All the controls were tested for aspartate transaminase (AST), alanine transaminase (ALT), CBC, HCV Ab, and serum ITIH4.

### 2.3. Liver Biopsy and Histopathological Evaluation

Liver biopsy was performed using an ultrasonography-guided true cut needle for all the patients. The mean length of the biopsy core provided was 1.16 ± 0.18 cm, ranging from 1.0 cm to 1.6 cm with a median of 1.0 cm. Specimens were fixed in formalin, embedded in paraffin, and stained with hematoxylin and eosin, Masson's trichrome, reticulin, and Perl's stains. Hepatic necroinflammatory activity and liver fibrosis were evaluated according to Ishak staging and grading score. Necroinflammatory activity was classified into minimal (score 1–3), mild (score 4–8), moderate (score 9–12), and severe (score 13–18) [19]. Fibrosis was classified into mild (stage 1), moderate (stages 2-3), and severe fibrosis or cirrhosis (stages 4–6) [5]. Significant fibrosis was defined as Ishak score of 3 or more (presence of bridging fibrosis) [20]. AST-to-platelet ratio index (APRI) and FIB-4 index were calculated according to the formula APRI = AST/upper limit of normal × 100/platelet count (10^9^/L), FIB-4 = Age (years) × AST/platelet count (10^9^/L) × (ALT)^1/2^ [21] and compared in different stages of fibrosis.

### 2.4. Statistical Analysis

Descriptive results were expressed as mean ± standard deviation (SD) or number (percentage) of individuals with a condition. For quantitative data, statistical significance was tested by Mann-Whitney *U* nonparametric test. For qualitative data, significance between groups was tested by Chi-square test. Correlation was tested by Spearman's test. Results were considered significant if *P* value ≤ 0.05. Statistical analysis was performed using SPSS statistical package version 13 (SPSS Inc., Chicago, IL, USA).

## 3. Results

### 3.1. Study Population Characteristics

The study included 33 children with chronic HCV infection. They were 12 females and 21 males. Their mean age was 10.95 ± 4.53 ranging from 3.5 to 18 years. A second group of 30 age- and sex-matched (*P* > 0.05 for both) healthy children served as controls. They were 11 females and 19 males. Their mean age was 11.13 ± 4.04 ranging from 4 to 17 years. The major possible modes of infection were male circumcision (63.6%) followed by presence of a family member with HCV infection (60.6%), surgery (42.42%), blood transfusion (27.27%), and dental procedures (15.15%). Many children had more than one possible mode of infection. The majority of patients (87.9%) were asymptomatic while four (12.1%) children presented with abdominal enlargement. Clinically, four (12.1%) children had hepatomegaly, one child (3.0%) had splenomegaly, and none had jaundice or ascites. Fibrosis stage ranged from F1 to F3 and activity grade ranged from A1 to A8. The majority of patients (84.8%) had either F1 or F2 fibrosis and mild activity (66.7%), and 8 out of 33 (24.2%) had steatosis (Table 1).

### 3.2. Histopathological Findings in Patients with Normal versus Elevated Transaminases

All the patients had mild to moderate fibrosis and minimal to mild activity in liver biopsy. Yet, nearly half of them had normal transaminases (46.2%, 45.0%, 45.5%, and 45.5% with mild fibrosis, moderate fibrosis, minimal activity, and mild activity, resp.) (Table 2).

### 3.3. ITIH4 according to Disease Severity and Correlation with Laboratory and Histopathological Parameters

The mean value of serum ITIH4 was significantly higher in patients than in controls (54.2 ± 30.78 pg/mL versus 37.21 ± 5.39 pg/mL; *P* = 0.021). There was no statistical significant difference in the mean level of ITIH4 when comparing patients with different activity grades and patients with normal transaminases versus those with elevated transaminases (*P* > 0.05 for both; Table 3) but it was slightly higher in those with moderate fibrosis and in those with elevated transaminases. In addition, there was a significant direct correlation between ITIH4 and the stage of fibrosis (*P* = 0.015) while there was no significant correlation with the other studied laboratory parameters, yet, the negative correlation with HCV viremia was of marginal significance (*P* = 0.071) (Table 4). On the other hand, there was no correlation between APRI (*r* = −0.009 and *P* = 0.961) and FIB-4 (*r* = 0.155 and *P* = 0.389) with the stage of fibrosis.

### 3.4. ITIH4, but Not APRI or FIB-4 Is Significantly Higher in Patients with Significant Fibrosis (Ishak Score ≥ 3)

**Figure 1:**
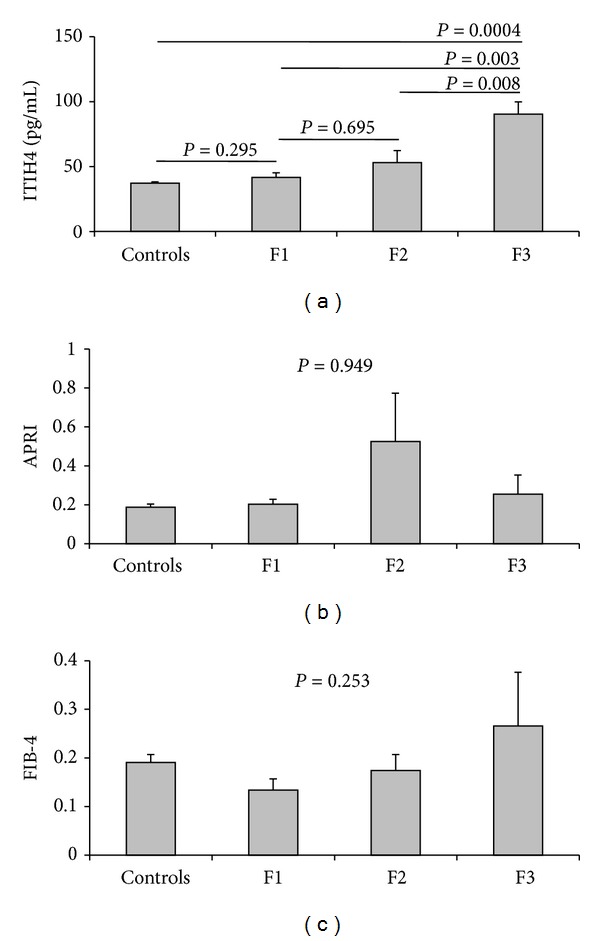
Serum fibrosis markers in the individual fibrosis stages. (a) ITIH4; (b) APRI; and (c) FIB-4.

Serum ITIH4 was at its lowest (41.63 ± 13.15 pg/mL) in patients with F1, increasing in patients with F2 (53.03 ± 35.75 pg/mL) and reaching the highest level in F3 (90.42 ± 20.67 pg/mL). Though the levels in F1 and F2 had no significant difference from those in healthy controls, the levels in F3 were significantly higher than those in F1, F2, and the controls (Figure 1(a)). On the other hand, there was no statistically significant difference in the mean values of APRI (*P* = 0.949) and FIB-4 (*P* = 0.253) according to different stages of fibrosis, yet, FIB-4 tended to increase with higher stages of fibrosis (Figures 1(b) and 1(c), resp.).

### 3.5. ITIH4 according to Hepatitis C Viral Load

**Figure 2:**
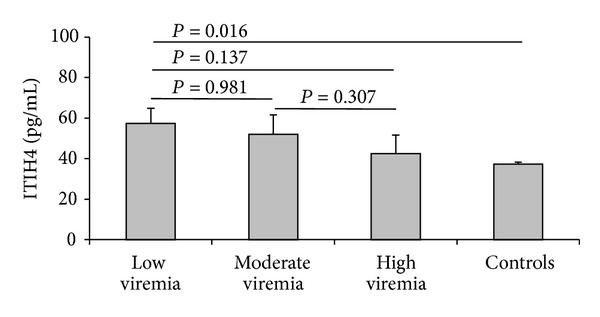
Serum ITIH4 in different levels of HCV viremia.

Serum ITIH4, though not statistically significant (*P* = 0.356), was negatively associated with the level of viremia. It was higher (57.29 ± 34.13 pg/mL) in patient with low viremia trending to be successively lower in patients with moderate and low viremia (51.95 ± 27.41 pg/mL and 42.51 ± 18.03 pg/mL resp.). The levels in patients with low viremia were significantly higher compared to that in controls (*P* = 0.016) (Figure 2).

## 4. Discussion

The natural history of chronic hepatitis C in children differs from that in adults since HCV infection is relatively benign, induces mild changes in the liver with a low level of fibrosis and a low rate of progression, and is rarely associated with severe or decompensate liver disease [22]. Bortolotti et al. [23] reported that hepatitis C in children is usually asymptomatic. Most of our patients (87.9%) were asymptomatic, while the other patients sought medical advice because of abdominal enlargement. Clinically, 4 (12.1%) had hepatomegaly and only one had splenomegaly but none had jaundice or ascites. A similar finding was reported by El-Raziky et al. [5] since soft enlargement of the liver was found in two (11%) children with HCV infection and none had splenomegaly or ascites.

In the current study, although all the patients had mild to moderate fibrosis and minimal to mild activity in liver biopsy, nearly half of them had normal transaminases. It has been reported that alanine transaminase levels were normal in half of the subjects, yet, histological abnormalities were detectable in three quarters of HCV-RNA positive cases [5]. This means that liver enzymes in chronic HCV infection do not necessarily reflect the histopathological abnormalities in the majority of cases and liver biopsy would be essential for evaluation of the disease state and extent of liver injury.

APRI and FIB-4 have been of interest to clinicians because they are simple to calculate and readily available from hospital or clinic laboratories during usual patient care [21]. In our study, APRI and FIB-4 showed no statistically significant difference (*P* > 0.05 for both) among fibrosis stages; however, FIB-4 tended to increase successively from F1 to F3. In hand with our results, Díaz et al. [24] reported that APRI significantly predicts cirrhosis but not fibrosis in pediatric patients. de Lédinghen et al. [25] found that APRI was of benefit in predicting cirrhosis in children with various chronic liver diseases. The majority of reports using APRI and FIB-4 showed a significant performance in discriminating F0–F2 from F3-F4 Metavir score [21], or discriminating F0–F3 from F4–F6 Ishak score [26]. Such advanced stages of fibrosis or cirrhosis were not detected in our study population.

APRI and FIB4 reflect alterations in hepatic functions rather than in extracellular matrix (ECM) metabolism. Since, several HCV reports have described normal transaminase levels in about 25%–30% of chronic HCV patients, there may be a potential advantage in assessing serum direct fibrosis markers that do not involve transaminases [27], of which ITIH4 can be considered as one. In our study, 45.5% (15/33) of patients had normal transaminases despite the presence of mild or moderate fibrosis. This may explain the unsatisfactory results of APRI and FIB-4 for detection of significant fibrosis in our study compared to that of ITIH4.

There have been many studies on the biological effects of the ITI molecules, proposing an involvement in various acute-phase processes such as inflammation and cancer [28]. Inhibition of tumor growth and spreading mediated by ITIH genes most likely relates to their stabilizing effects on the extracellular matrix, as well as their covalent linkage of hyaluronic acid [29]. The anti-inflammatory role for ITI has been suggested by the discovery of stable complexes between these proteins and tumor necrosis factor-stimulated gene 6 [30].

The main target of the current study was to evaluate serum ITIH4 level in children with chronic HCV infection and its relation to liver fibrosis and the level of viremia. Our results showed that ITIH4 serum levels increased successively as fibrosis progresses from F1 to F3. This is in agreement with Yang et al. [10] who reported that ITIH4 was in its lowest value in Metavir F1 and increased as fibrosis progresses then decreased at F4 (cirrhosis). In addition, Gangadharan et al. [11] reported that ITIH4 appears to be candidate to discriminate Metavir F3 from F4 fibrosis.

In patients with no or minimal fibrosis at presentation, antiviral treatment could possibly be delayed due to the mild nature of the disease and the slow progression of liver fibrosis, while in those with significant fibrosis, antiviral treatment is a priority [31]. For that, identifying patients with significant fibrosis is of utmost importance. In the current study, ITIH4 was significantly higher in patients with significant fibrosis (F3) than in those with lower fibrosis stages.

Liver fibrosis represents a chronic wound repair following diverse insults. In fibrogenesis, the normal ECM deposition in the space of Disse is switched to fibrillar, contractile ECM. Thus, it is reasonable to speculate that a proteolytic degradation of the normal ECM may occur at the onset of liver fibrogenesis by matrix metalloproteinases (MMPs). MMP-2 and MMP-9 are the most impressively induced matrix metalloproteinases [32]. Furthermore, the multifunctional cytokine transforming growth factor-beta (TGF-*β*) plays a pivotal role in the occurrence and progression of fibrosis to cirrhosis. TGF-*β* enhance hepatocyte destruction and mediate hepatic stellate cell and fibroblast activation resulting in a wound healing response, including myofibroblast generation and ECM deposition [33].

Trypsin activates both MMP-2 [34] and MMP-9 [35]. In addition, the activation of pro-MMP-9 is inhibited by the human trypsin inhibitor [36]. Protease inhibitors prevent the induced hepatic fibrosis in rats through profound inhibition of TGF-*β* generation [37]. So, the increased ITIH4 with higher stages of fibrosis may be an attempt of the body to counteract the fibrogenic process mediated by the upregulated inflammatory cytokines and MMPs.

ITIH4 is one of the structurally related serine protease inhibitors [13]. The approval of the new direct inhibitors of HCV replication, protease inhibitors, reflects a major advance for patients infected with HCV [38]. HCV viremia is a determinant factor for the treatment customization and outcomes where low viral load was found to be associated with favourable response [39]. The main target of these direct acting antivirals is to antagonize critical viral proteins including nonstructural protein 3 (NS3) and NS3/4A serine proteases which are important for viral replication [40, 41]. This results in decreased viral load, paving the way for the action of the standard regimen of therapy (pegylated interferon/ribavirin) [42].

We found that HCV viremia was lower in patients with higher serum ITIH4 levels and increases as ITIH4 decreases with highest viremia in patients with lowest ITIH4 levels. It was reported that influenza virus is activated upon trypsin treatment [43] and protease inhibitors were found to inhibit influenza virus* in vitro* [44]. Furthermore, human pancreatic trypsin inhibitor was found to inhibit the NS3 protease of HCV [45]. Worth to mention, ITIH4 is predominantly expressed in the liver and pancreas [28]. Taken together, it would be logical to postulate that ITIH4 may have potential antiviral properties against HCV.

The small number of the study population represents a limitation which cannot be easily overcome in a single center study. This preliminary work needs to be confirmed in a larger population. In addition, it is worthwhile to evaluate the diagnostic performance of ITIH4 in other pediatric liver diseases with more advanced fibrosis or even cirrhosis.

## 5. Conclusion

In conclusion, our study demonstrated that ITIH4 serum levels were associated with higher stages of liver fibrosis and were significantly higher in patients with significant fibrosis than in those with lower fibrosis stages. The trend of higher ITIH4 levels in those with lower viral loads suggests potential antiviral properties of ITIH4 and further studies to investigate its effect on HCV replication are worthwhile.

## Figures and Tables

**Table 1:** Laboratory and histopathological characteristics of the studied patients.

Parameter	HCV patients (*n* = 33)
Liver function tests	
Total bilirubin (mg/dL)	0.98 ± 0.39
Direct bilirubin (mg/dL)	0.29 ± 0.25
Albumin (g/dL)	4.25 ± 0.72
Alanine transaminase (U/L)	69.45 ± 125.89
Aspartate transaminase (U/L)	52.79 ± 120.49
Gamma glutamyl transpeptidase (U/L)	46.38 ± 16.38
Alkaline phosphatase (U/L)	257.79 ± 94.92
Fibrosis stage	
F1	13 (39.4)
F2	15 (45.4)
F3	5 (15.2)
Activity grade	
Minimal (A1–A3)	11 (33.3)
Mild (A4–A8)	22 (66.7)
Steatosis	8 (24.2)

**Table 2:** Histopathological findings in patients with normal versus elevated transaminases.

Histopathology	Normal transaminases *n* = 15	Elevated transaminases *n* = 18	*P* value
Fibrosis stage			
Mild fibrosis (*n* = 13)	6 (46.2)	7 (53.8)	0.948
Moderate fibrosis (*n* = 20)	9 (45.0)	11 (55.0)	
Activity grade			
Minimal activity (*n* = 11)	5 (45.5)	6 (54.5)	1.0
Mild activity (*n* = 22)	10 (40)	12 (54.5)	

**Table 3:** Serum ITIH4 according to disease activity.

Parameter	ITIH4 (pg/mL)	*P* value
Activity grade		
Minimal (*n* = 11)	47.17 ± 14.36	0.566
Mild (*n* = 22)	57.72 ± 36.15	
Transaminases level		
Normal (*n* = 15)	53.02 ± 25.27	0.856
Elevated (*n* = 18)	55.19 ± 35.43	

**Table 4:** Correlation of ITIH4 with laboratory and histopathological parameters in liver biopsy.

Parameter	ITIH4 (pg/mL)	
	*r*	*P*-value
Total bilirubin (mg/dL)	−0.233	0.193
Direct bilirubin (mg/dL)	−0.204	0.254
Albumin (g/dL)	−0.232	0.194
Alanine transaminase (U/L)	−0.03	0.867
Aspartate transaminase (U/L)	−0.141	0.434
Gamma glutamyl transpeptidase (U/L)	−0.316	0.684
Alkaline phosphatase (U/L)	−0.143	0.506
HCV-RNA (IU/mL)	−0.318	0.071
Fibrosis stage	0.422	0.015
Activity grade	0.082	0.650
